# Propensity score-matched analysis for comparing transpancreatic sphincterotomy and needle-knife precut in difficult biliary cannulation

**DOI:** 10.1038/s41598-021-84655-2

**Published:** 2021-03-15

**Authors:** Fatema Tabak, Fei Wang, Guo-Zhong Ji, Lin Miao

**Affiliations:** Institute of Digestive Endoscopy and Medical Center for Digestive Diseases, The Second Affiliated Hospital of Nanjing Medical University, Nanjing, 210011 Jiangsu China

**Keywords:** Gastroenterology, Medical research

## Abstract

Transpancreatic sphincterotomy (TPS) can be an alternative approach of difficult biliary access in endoscopic retrograde cholangiopancreatography (ERCP). We aimed to evaluate the efficacy and safety of TPS compared to needle-knife precut (NKP), considering the early and late outcomes of both techniques. The prospectively collected clinical data, ERCP procedure findings, and outcomes of patients who underwent ERCP with difficult biliary access in our hospital from July 2016 to January 2018 were retrospectively analyzed. The patients were divided into two groups according to the applied secondary cannulation techniques. The propensity score matching (PSM) was applied to reduce the potential selection bias and unify the preventive measures of post-ERCP pancreatitis (PEP) in both groups. A total of 125 patients were enrolled in this study, with 54.4% male and a mean age of 63.29 ± 16.33 years. NKP group included 82 patients, and 43 patients received TPS. Prophylactic pancreatic stents were placed in all patients with TPS and 58.5% of patients with NKP. After applying PSM, the cohort was comprised to 86 patients with 43 patients in each TPS and NKP groups. Successful selective cannulation was achieved by 95.3% using TPS and by 93% using NKP. The mean procedure time was shorter in the TPS group without significant difference. Compared to NKP, using TPS did not affect the rate of PEP. Moreover, TPS was associated with less frequent post-ERCP bleeding and perforation, but without significant differences (all p > 0.05). Patients who received TPS or NKP had no symptoms related to papillary stenosis or chronic pancreatitis during the follow-up period. In conclusion, using TPS in difficult cannulation cases was useful to achieve success cannulation with an acceptable PEP rate and less frequent post-ERCP bleeding and perforation compared to NKP. There were no symptoms related to papillary stenosis or chronic pancreatitis during the follow-up period.

## Introduction

Endoscopic retrograde cholangiopancreatography (ERCP) has become a common therapeutic intervention for several pancreaticobiliary conditions. Selective cannulation of the common bile duct (CBD), which is the key to the successful biliary therapeutic procedure, could be achieved after a few attempts of standard cannulation methods in around 80% of cases^1^.

According to the European Society of Gastrointestinal Endoscopy (ESGE) guidelines, biliary cannulation is defined as difficult if cannulation lasts longer than five minutes, success requires more than five attempts, or the guidewire accidentally passes the pancreatic duct at least twice. Therefore, additional cannulation methods are often needed in difficult cannulation cases. Difficult cannulation is frequently reported as a risk factor for adverse events with a probability of failed biliary cannulation ranges from 5 to 18% of cases^1–3^.

Different techniques are reported in the literature regarding papillary cannulation in difficult cases^4–7^. Transpancreatic sphincterotomy (TPS) is a technique used for exposing the bile duct orifice by making an incision through the septum between the pancreatic and biliary duct. It involves the placement of a papillotome in the pancreatic duct and performing sphincterotomy in the direction of the bile duct, then extending the sphincterotomy to cannulate the biliary duct^1,8,9^. TPS is resorted when attempts with the primary methods had failed and should be performed by experienced endoscopists.

ERCP is an invasive procedure with an overall adverse events rate of approximately 4–11%, and the most common one is post-ERCP pancreatitis (PEP)^10,11^. Using advanced cannulation techniques in a native papilla is considered a risk factor of PEP. The ESGE suggested prophylactic pancreatic stenting in patients receiving TPS to decrease the risk of PEP^1,12,13^. Pancreatic duct stricture or chronic pancreatitis could be developed after pancreatic sphincterotomy; therefore, a long follow-up period is needed to detect those adverse outcomes.

Several retrospective studies compared the effects of different cannulation techniques on ERCP outcomes, but without considering uniformed PEP preventive measures^4,14,15^. Therefore, more studies are needed to clarify the role of TPS in the case of difficult biliary access. This study tries to fill the gap and offer a new evaluation of the efficacy and adverse events rate of TPS compared to NKP, considering the same PEP preventive methods in both techniques. Furthermore, we aim to evaluate the impact of TPS in developing any symptoms related to the ductal stricture or chronic pancreatitis (CP) during the 6-month follow-up period.

## Methods

### Data source and participants

**Figure 1:**
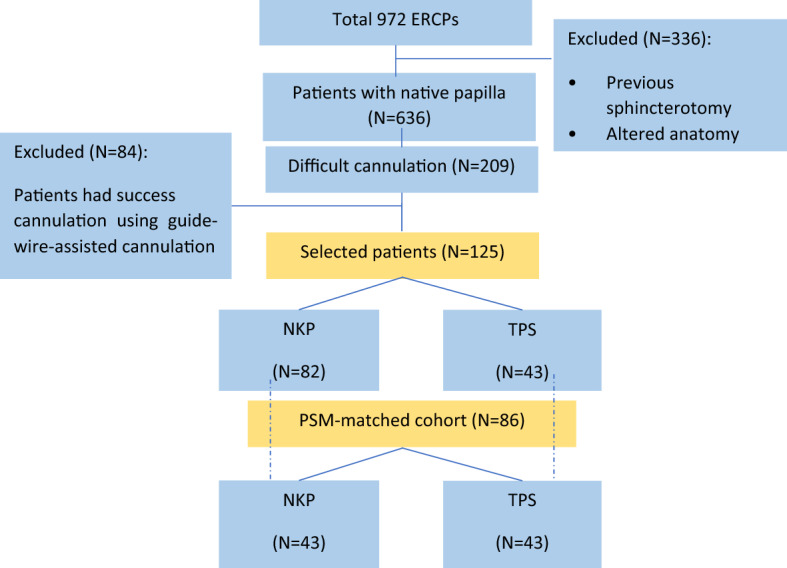
Study design and sample breakdown based on used difficult cannulation techniques before and after propensity score matching (PSM).

The study protocol was approved by the institutional review board of the Second Affiliated Hospital of Nanjing Medical University. The clinical data of 972 patients who underwent ERCP in our hospital between July 2016 and January 2018 were prospectively collected, and all patients gave their written informed consent to participate in ERCP after full explanation of the procedure. The clinical procedures were carried out in accordance with the Declaration of Helsinki. After excluding the patients with previous sphincterotomy and patients with altered anatomy, the difficult cannulation was reported in 209 cases (see Fig. 1), and these cases were retrospectively analyzed to achieve the aim of this study.

In 84 difficult cannulation cases, repeating guidewire-assisted cannulation attempts was a successful method to achieve deep cannulation. Accordingly, a total of 125 difficult cannulation cases required using NKP or TPS during ERCP to achieve biliary cannulation. For the purpose of analysis, those 125 patients were divided into two groups based on the secondary cannulation technique. Patients' clinical and therapeutic features, including demographics, indications, procedure details, and ERCP related adverse events, were analyzed. Additionally, comorbidities were weighted using the Charlson Comorbidity Index (CCI) depending on the risk of mortality associated with each comorbid disease. We used the cutoff of CCI ≥ 2 to stratify the patients based on their comorbidities.

### ERCP procedure

All ERCP procedures were performed by three experienced endoscopists in our center with an experience of over 250 ERCPs/year in the last 5 years. Patients underwent therapeutic ERCP using standard side-view duodenoscope following an overnight fast under conscious sedation. Patients were monitored continuously during the procedure using a pulse oximeter, electrocardiography monitoring, and an automatic blood pressure recording device. Supplementary oxygen was provided when needed. Prophylactic octreotide dose was administered to all patients before ERCP for PEP prevention. Besides, a prophylactic pancreatic stent (PPS) was deployed in difficult cannulation cases, especially with multiple pancreatic cannulations.

**Figure 2:**
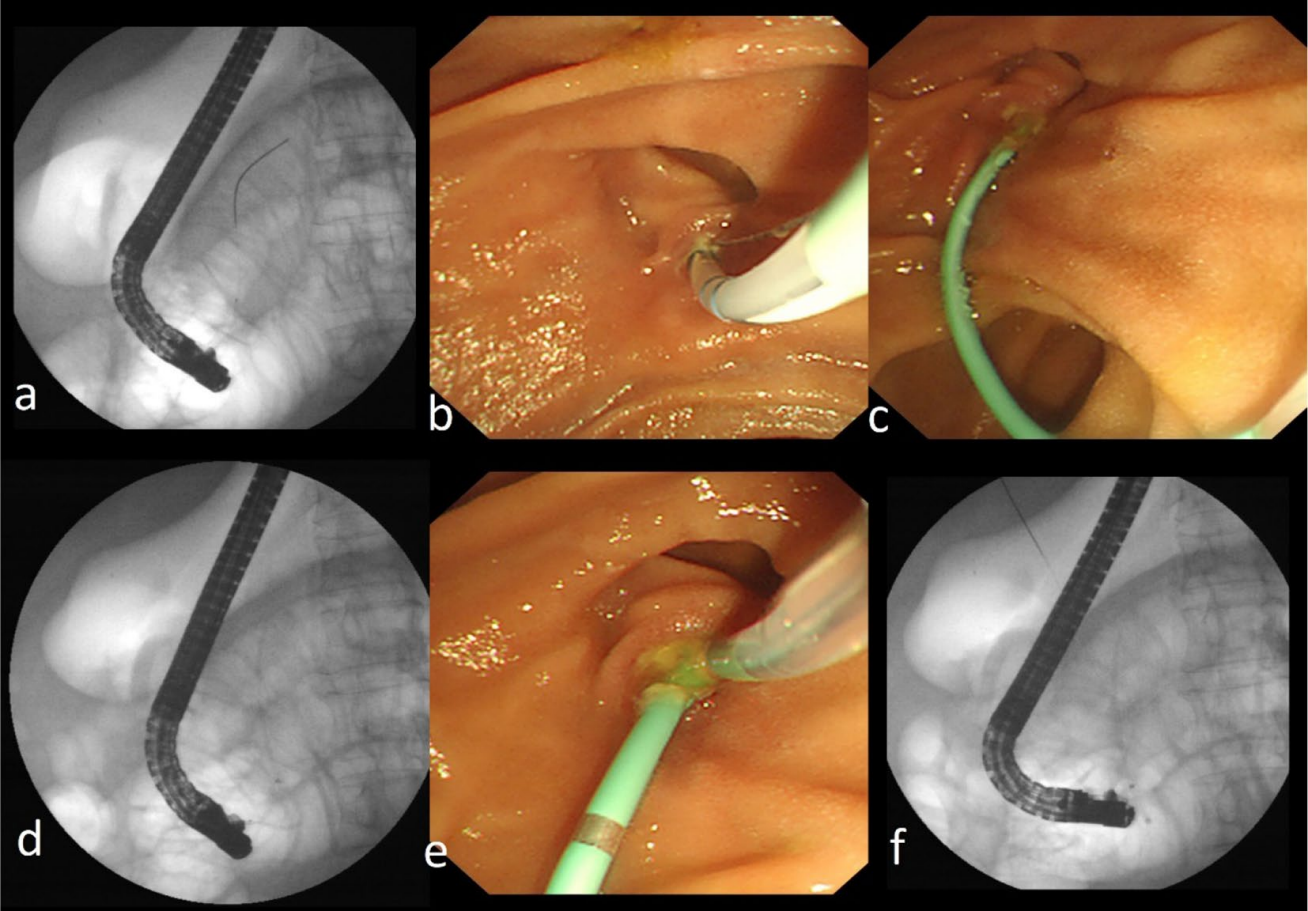
Transpancreatic sphincterotomy technique. (**a**) Fluoroscopy image showed the guidewire inserted in the pancreatic duct. (**b**) The septum was cut with a sphincterotome from the pancreatic duct towards the bile duct axis. (**c**) The placement of pancreatic duct stent. (**d**) After a stent was placed in the pancreatic duct. (**e**) Cannulation toward the bile duct was performed. (**f**) The biliary cannulation was successfully achieved.

Initial biliary cannulation was routinely performed using a guidewire-assisted technique with sphincterome. Needle knife precut or transpancreatic sphincterotomy was resorted when selective biliary access failed after standard cannulation attempts. Both conventional precut sphincterotomy and needle knife fistulotomy were used to apply the precut method in difficult cannulation cases. Choosing the suitable secondary cannulation technique was according to the expertise of endoscopists, which was mainly based on the orientation and morphology of the papilla and the repetitive unintended guidewire insertion into the pancreatic duct. NKP was considered as the preferred choice in the absence of pancreatic cannulation and the cases with impacted biliary stones at the ampulla or the distal CBD. In the cases of prior guidewire insertion in the pancreatic duct, TPS was performed using a sphincterotome towards the bile duct axis. After that, the pancreatic stent was placed, then the biliary cannulation was performed (see Fig. 2). An additional needle-knife incision was applied in some cases from the upper end of the previous pancreatic sphincterotomy towards 10 o'clock to expose the bile duct.

The standard process in our hospital requires that all the patients should be hospitalized 3 days after the procedure for observation and serum amylase testing after the procedure. All discharged patients were informed to stay in contact for any delayed adverse events.

### Definitions and criteria

According to ESGE criteria^1^, cannulation was considered difficult if either took more than 5 min, if it needed more than five cannulation attempts on the papilla, or if the pancreatic duct was cannulated more than one time.

All adverse events were defined according to published criteria. Post-ERCP pancreatitis was defined as new or worsened abdominal pain with an elevated amylase at least three times the upper limit of the normal level, at more than 24 h after ERCP, requiring admission or prolongation of planned hospitalization. Post-procedural bleeding evidenced by a drop-in haemoglobin > 2 g/dl. Perforation was diagnosed according to the imaging evidence of intraperitoneal or retroperitoneal leakage of contrast agent observed under radioscopy.

### Data analysis

Differences among different patient were determined by using Fisher's exact test for categorical variables, and non-categorical variables with the Mann–Whitney U test. Univariate regression was performed with significant variables (p < 0.05). To balance clinical variables between NKP and TPS patients, we used multivariable logistic regression to generate propensity scores for all match-eligible patients. Propensity score matching (PSM) analysis^16^ is performed to reduce the potential bias in this observational study, achieving a more fair comparison between the two groups regarding PEP prevention measures. The propensity score of each patient was calculated by the logistic regression model; the cannulation method was set as the dependent variable, co-variables including age, sex, and using PPS. We used a one-to-one match strategy with the nearest-neighbour matching algorithm without replacement to obtain PSM-matched samples. The balance of the two groups was assessed using standardized differences. The MATCHIT package with R (version 3.6) was used to perform PSM. All statistical analyses were performed using SPSS statistics version 23 for Windows.

### Institutional review board statement

This study was reviewed and approved by the Ethics Committee of the Second Affiliated Hospital of Nanjing Medical University.

### Informed consent statement

All study participants, or their legal guardian, provided informed written consent before study enrollment.

### STROBE statement

The authors have read the STROBE Statement-checklist of items, and the manuscript was prepared and revised according to the STROBE Statement-checklist of items.

## Results

### Patient characteristics of the total cohort

A total of 125 patients with difficult cannulation were enrolled, including 68 males (54.4%) with a mean age of 63.29 ± 16.33 years. Among them, 82 patients (65.6%) required using needle-knife precuts, so they were grouped as NKP group, and 43 patients (34.4%) received transpancreatic sphincterotomy in TPS group. The characteristics and indications for ERCP are summarized in Table 1. There was no significant difference in the median age or patients' gender between NKP and TPS groups, with a male percentage (52.4% vs. 58.1%, p = 0.338). No significant difference was noted in overall comorbidities and the proportion of patients with (CCI) ≥ 2 between the two groups (30.4% vs. 18.6%, p = 0.343). The majority of the procedures were performed due to biliary stones in both groups (65.8% vs. 67.4%, p = 0.511), without any significant difference in the indication distribution between NKP and TPS groups.

**Table 1:** Characteristics' summary and indications for ERCP in the total cohort and PSM-matched groups.

Characteristics	Entire cohort (n = 125)			PSM cohort (n = 86)		
	NKP (n = 82)	TPS (n = 43)	P value	NKP (n = 43)	TPS (n = 43)	P value
Age [mean ± standard deviation] (year)	68.36 ± 14.95	59.43 ± 17.40	0.234	61 ± 15.30	59.43 ± 17.40	0.822
Charlson score ≥ 2	30.4% (25)	18.6% (8)	0.110	27.9% (12)	18.6% (8)	0.222
Male	52.4% (43)	58,1% (25)	0.338	60.4% (26)	58,1% (25)	0.500
**Indications**						
Biliary stones	65.8% (54)	67.4% (29)	0.511	65.1% (28)	67.4% (29)	0.500
Benign strictures	21.9% (18)	23.2% (10)	0.518	18.6% (8)	23.2% (10)	0.396
Cholangiocarcinoma	3.6% (3)	6.9% (3)	0.339	2.3% (1)	6.9% (3)	0.308
Cholangitis	6.1% (5)	2.3% (1)	0.325	2.3% (1)	2.3% (1)	0.753
Biliary pancreatitis	2.4% (2)	6.9% (3)	0.222	4.6% (2)	6.9% (3)	0.500
Ampullary carcinoma	3.6% (3)	2.3% (1)	0.573	4.6% (2)	2.3% (1)	0.500
Pancreatic cancer	12.2% (10)	6.9% (3)	0.281	13.9% (6)	6.9% (3)	0.242

### PSM grouping

Since prophylactic pancreatic stent was applied to all patients who received the TPS technique and just for patients with multiple pancreatic duct access in the NKP group, there was a significant difference between both groups regarding PPS placement (p < 0.001). In order to unify the PEP prevention strategy and to reduce the potential selection bias in both groups, we performed the propensity score matching. We have matched TPS and NKP cohorts regarding age, gender, and PPS placement.

The changes in the clinical data after rearranging the two PSM-matched groups were shown in Table 1. The propensity score-matched cohort included 51 males (59.3%), with a median age of 60.21 ± 16.31 years for the PSM cohort. Both PSM-matched NKP and TPS groups have 43 patients with comparable median age and gender without a significant difference in the indication distribution.

### The success rate of biliary cannulation in PSM-matched groups

**Figure 3:**
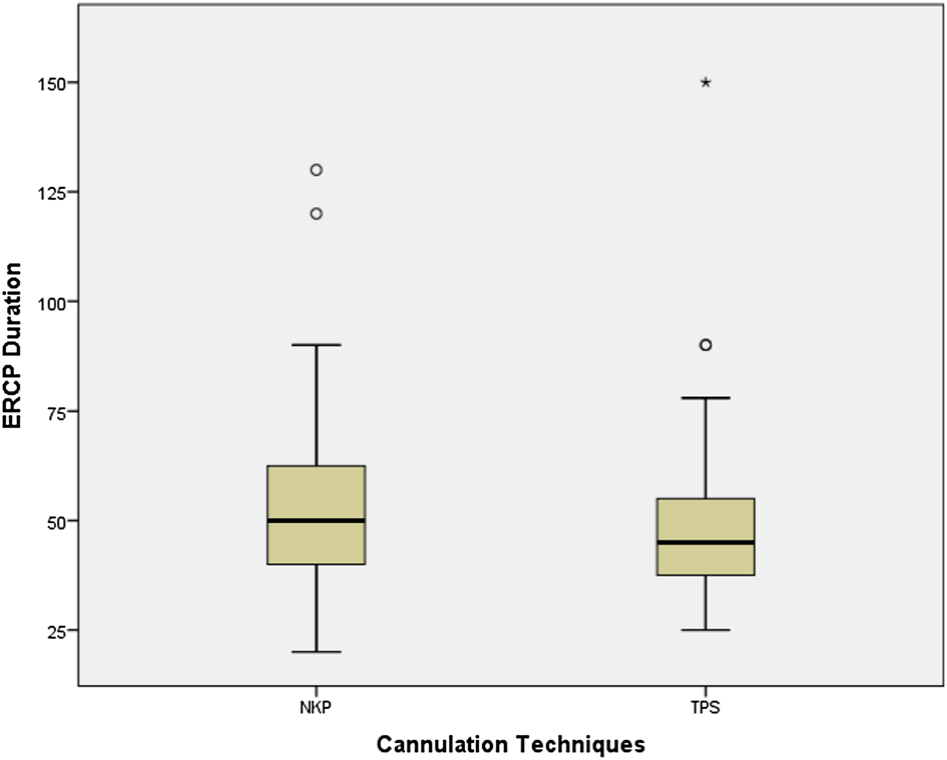
ERCP duration in NKP and TPS cannulation techniques for the enrolled patients. The box is shorter for TPS than NKP. The inner fences extend less for TPS compared to NKP. That is, the procedure time varies less for TPS than for NKP.

Table 2 reports the details of cannulation and procedure after successful cannulation in the total cohort and PSM-matched groups. The cannulation success rate did not differ among groups (p = 0.500). The overall success rate of biliary cannulation was 93% (40/43) with NKP, 95.3% (41/43) with TPS. TPS was used more commonly in the case of the small papilla, while NKP was frequently used for protruding papilla, but without any significant difference in the duodenal papilla morphology distribution between NKP and TPS groups. Both groups were comparable regarding the mean cannulation time (p = 0.113). However, using TPS was associated with shorter procedure duration (49.79 ± 21.88) minutes comparing with (53.38 ± 22.71) minutes in the NKP group but did not reach a significant difference (see Fig. 3). After successful cannulation, no statistically significant differences were noted in the additional therapeutic interventions among the two groups.

**Table 2:** Details of cannulation and procedures after successful cannulation in the total cohort and PSM-matched groups.

Characteristics	Entire cohort (n = 125)			PSM cohort (n = 86)		
	NKP (n = 82)	TPS (n = 43)	P value	NKP (n = 43)	TPS (n = 43)	P value
ERCP procedure time (mean) (min)	53.54 ± 27.55	49.79 ± 21.88	0.395	53.38 ± 22.71	49.79 ± 21.88	0.460
Cannulation time (mean) (min)	10.41 ± 1.43	10.44 ± 1.02	0.475	10.69 ± 0.68	10.44 ± 1.02	0.113
Cannulation success rate	94% (77)	95.3% (41)	0.529	93% (40)	95.3% (41)	0.500
**Duodenal papilla morphology**						
Small papilla	18.3% (15)	27.9% (12)	0.938	20.9% (9)	27.9% (12)	0.582
Regular papilla	31.7% (26)	34.9% (15)	0.645	32.5% (14)	34.9% (15)	0.276
Protruding papilla	23.1% (19)	14% (6)	0.227	25.5% (11)	14% (6)	0.108
Peripapillary diverticulum	26.8% (22)	25.5% (11)	0.529	20.8% (9)	25.5% (11)	0.139
**Procedures after successful cannulation**						
EPBD	67.1% (55)	62.2% (27)	0.764	68.7% (55)	62.2% (27)	0.437
Biliary stent placement	25.6% (21)	25.5% (11)	0.588	21.4% (11)	25.5% (11)	0.554
ENBD	53.6% (44)	53.5% (23)	0.963	55% (44)	53.5% (23)	0.722
Biliary stone removal	62.2% (51)	65.1% (28)	0.674	63.7% (51)	65.1% (28)	0.644
Stricture dilatation	13.4% (11)	13.9% (6)	0.253	13.7% (11)	13.9% (6)	0.212
Pancreatic stent placement	58.5% (48)	100% (43)	p < 0.001	100% (43)	100% (43)	0.753

*EPBD* endoscopic sphincterotomy with balloon dilation, *ENBD* endoscopic nasobiliary drainage.

### Adverse events in PSM-matched groups

Table 3 reports the ERCP-related short-term outcomes in the total cohort and PSM-matched groups. The overall adverse events occurred in (8/86) patients (9.5%) of the PSM-matched population. They were more frequent after NKP, including five patients (11.6%) compared with three patients (7%) in the TPS group, but without a significant difference (p = 0.227). The most common adverse event in both groups was PEP, and no significant difference was noted regarding its occurrence in both groups. Post-procedure hyperamylasemia was more frequent in the TPS group (23.2%) compared with the NKP group (13.6%), but no significant difference was observed after PSM matching (p = 0.071).

**Table 3:** ERCP-related short-term outcomes in the total cohort and PSM-matched groups.

Characteristics	Entire cohort (n = 125)			PSM cohort (n = 86)		
	NKP (n = 82)	TPS (n = 43)	P value	NKP (n = 43)	TPS (n = 43)	P value
Adverse events	10.9% (9)	7% (3)	0.495	11.6% (5)	7% (3)	0.227
PEP	4.8% (4)	7% (3)	0.455	4.6% (2)	7% (3)	0.5
Perforation	2.4% (2)	0% (0)	0.429	2.3% (1)	0% (0)	0.247
Bleeding	2.4% (2)	0% (0)	0.279	2.3% (1)	0% (0)	0.247
Cholangitis	1.2% (1)	0% (0)	0.279	2.3% (1)	0% (0)	0.5
Hyperamylasemia	8.7% (7)	23.2% (10)	0.025	13.6% (6)	23.2% (10)	0.071
Duration of hospitalization [median (IQR)] (day)	8.41 ± 7.01	8.36 ± 5.59	0.937	7.60 ± 7.36	8.36 ± 5.59	0.348
Second ERCP	12.2% (8)	13.9% (6)	0.339	11.6% (5)	13.9% (6)	0.453

*IQR* interquartile range.

The incidence of procedure-related bleeding and perforation did not significantly differ among the two groups, although the two bleeding and two perforation cases were recorded in the NKP group before PSM matching. Both two bleeding cases were managed with endoscopic submucosal injection and/or placement of hemostatic clips, and blood transfusion when needed without surgical intervention. The two perforations cases were identified immediately at the time of ERCP and treated conservatively for around 10-days hospitalization with no requirement for surgical intervention. The patients' hospitalization duration was comparable in both groups without significant difference (p = 0.384).

### Long-term outcomes in the total cohort

All patients who enrolled in the NKP and TPS groups were followed during 6 months after the procedure in the outpatient's department to assess any long-term adverse events. During this period, patients in both groups had no symptoms related to papillary stenosis, pancreatic strictures, or chronic pancreatitis. No cholangitis relapse was reported; two patients in the NKP group and one in the TPS group had biliary stones recurrence. 12.2% of patients in the NKP group and 13.9% in the TPS group need the second ERCP to complete stones clearance, exchange or remove stents with no differences among groups. Two patients in the NKP group died during the follow-up period due to tumor invasion.

## Discussion

Success selective bile duct cannulation during ERCP can be achieved after a few attempts with standard guidewire-assisted cannulation in around 80% of cases. Recent guidelines recommended an early applying of advanced techniques in difficult canulation cases with the recommended insertion of prophylactic pancreatic stents when it is easy to obtain access to the pancreatic duct^1–3^. In our study, secondary cannulation was performed using needle-knife precut in 82 patients of all native papilla cannulation cases (12.8%) and using transpancreatic sphincterotomy in 43 patients (6.7%).

Regarding the efficacy of TPS, previous studies showed that TPS could be equally successful or even slightly better compared to other advanced cannulation methods in the case of difficult biliary access^15,17,18^. The overall cannulation success rate of TPS in our study was favourably close to those reported in a relevant meta-analysis, which reported a success rate of around 90% by different study designs^17,18^. Both TPS and KNP were effective in achieving successful biliary access without significant differences in the cannulation success rate and procedure duration.

In the relation between TPS and adverse events, our study reported that patients who received NKP had a higher frequency of overall adverse events compared to those who received TPS. However, there was no significant difference in the overall adverse events, as many recent studies suggested^14,15,18^.

Most studies showed that the PEP rate of TPS is similar to other advanced cannulation methods; even NKP could be better to avoid PEP^17,19,20^. However, limited studies have prospectively compared PEP rate in TPS and NKP, in addition to a lack of information on using uniform PEP preventive methods, which could make the PEP rate with TPS even lower^5,17^. The protective effect of the pancreatic stent in difficult cannulation cases has been strongly suggested by ESGE, and its insertion is not problematic since the guidewire is already in the pancreatic duct while performing TPS^1,17,19^.

In our study, the medical prevention of PEP was given to all patients before ERCP without using NSAID. In addition, we applied PPS for all patients undergoing TPS and for 58.5% of NKP patients, which was decided according to the guidewire passage into the pancreatic duct by the set time and not randomly determined. Thus, we performed the propensity score matching to achieve a more fair efficiency and safety comparison between NKP and TPS groups by excluding important factors that could influence PEP prevention's efficacy. Still, we noted an acceptable PEP rate in the two groups without any significant difference. In contrast, post-ERCP hyperamylasemia was more common in the group that received TPS comparing with NKP but did not reach a significant difference in PSM-matched groups. More frequent hyperamylasemia after using TPS could be related to the multi pancreatic guidewire insertion and the direct trauma to the pancreatic orifice because of several cannulation attempts.

As stated in a recent meta-analysis, the bleeding rate of TPS was in the range of (2–4%), which was accepted comparing with the approximate rate of (4%) for needle-knife precut techniques^17^. The perforation rate of TPS was remarkably low compared with other techniques in some reports^14,17^. In our study, no post-ERCP bleeding or perforation occurred after applying TPS, comparing with acceptable bleeding and perforation rate in the NKP group. That could be related to the wire-assisted method of TPS, which gives better control for cutting comparing with the freehand precut technique.

Only a few studies have evaluated the late adverse events of TPS^9,21^. Our follow-up found no patient developed symptoms related to papillary stenosis, ductal strictures, or chronic pancreatitis during 6 months after the procedure. Moreover, there was no significant difference related to recurrent biliary stones or cholangitis among the two groups.

Our study is performed at a single centre with a sample size likely limited the statistical differences in comparing the safety of TPS and NKP because of the rarity of adverse events. However, our sample size is still comparable with other studies in the literature, especially those focused on the difficult cannulation techniques. Therefore, larger samples for multi-center prospective studies with a more extended follow-up period are still required to evaluate the long-term risks of TPS. Despite this limitation, our study tried to fill the literature gap and provide supporting evidence about the success and safety of TPS using a prospectively recorded data with a 6-month follow-up period. Besides, we tried to unify the PEP prevention method to achieve a logical comparison between TPS and NKP.

In conclusion, this study has shown that TPS is a useful rescue method in difficult biliary cannulation when it is performed by experienced endoscopists. It is a well-tolerated technique and safe regarding early and late adverse events, with an acceptable PEP rate. Therefore, and similarly to ESGE guidelines, we suggest applying TPS in difficult cannulation cases with pancreatic stent placement.

## Data Availability

The datasets used and analyzed during the current study are available from the corresponding author on reasonable request.
